# Impact of deeper groundwater depth on vegetation and soil in semi-arid region of eastern China

**DOI:** 10.3389/fpls.2023.1186406

**Published:** 2023-06-29

**Authors:** Siteng Zhao, Xueyong Zhao, Yulin Li, Xueping Chen, Chengyi Li, Hong Fang, Wenshuang Li, Wei Guo

**Affiliations:** ^1^Northwest Institute of Eco-Environment and Resources, Chinese Academy of Sciences, Lanzhou, China; ^2^University of Chinese Academy of Sciences, Beijing, China; ^3^Naiman Desertification Research Station, Northwest Institute of Eco-Environment and Resources, Chinese Academy of Sciences, Tongliao, China; ^4^Tongliao Hydrology and Water Resources Sub-Center, Tongliao, China

**Keywords:** groundwater depth (GWD), successional stage (MD, SFD, FD), vegetation community characteristics, soil properties, ecological relationship, semi-arid region (Horqin Sandy Land)

## Abstract

**Introduction:**

Understanding the impact of deep groundwater depth on vegetation communities and soil in sand dunes with different underground water tables is essential for ecological restoration and the conservation of groundwater. Furthermore, this understanding is critical for determining the threshold value of groundwater depth that ensures the survival of vegetation.

**Method:**

This paper was conducted in a semi-arid region in eastern China, and the effects of deep groundwater depth (6.25 m, 10.61 m, and 15.26 m) on vegetation communities and soil properties (0–200 cm) across three dune types (mobile, semi-fixed, and fixed dunes) were evaluated in a sand ecosystem in the Horqin Sandy Land.

**Results:**

For vegetation community, variations in the same species are more significant at different groundwater depths. For soil properties, groundwater depth negatively influences soil moisture, total carbon, total nitrogen, available nitrogen, available phosphorus concentrations, and soil pH. Besides, groundwater depth also significantly affected organic carbon and available potassium concentrations. In addition, herb species were mainly distributed in areas with lower groundwater depth, yet arbor and shrub species were sparsely distributed in places with deeper groundwater depth.

**Discussion:**

As arbor and shrub species are key drivers of ecosystem sustainability, the adaptation of these dominant species to increasing groundwater depth may alleviate the negative effects of increasing groundwater depth; however, restrictions on this adaptation were exceeded at deeper groundwater depth.

## 1 Introduction

Water resources are scarce but essential in arid and semi-arid regions where a tight coupling exists between water resource availability, vegetation productivity, and energy circulation (Karthe, 2018; Ma et al., 2022). Alteration of water resource availability is the dominant factor in sustainability and restoration of vegetation communities and soil nutrients (Zeng et al., 2020; Condon et al., 2021; Reed et al., 2022). Both uptake from groundwater and soil water availability have been reported as the main mechanisms explaining the drought tolerance of vegetation (Huang et al., 2019; Barron-Gafford et al., 2021; Ma et al., 2022). For instance, Garrido found that *Prosopis tamarugo Phil.* survive in an arid region, which depends individually on the ability to extend roots to the groundwater in the almost rainless Atacama Desert (Garrido et al., 2016). Perez found that vegetation with ‘fast’ traits such as plant nitrogen concentration will rapidly obtain necessary resources, yet some vegetation with long life or poor resources may express inverse trends of ‘slow’ traits with a conservative resource use strategy (Perez-Harguindeguy et al., 2013). Indeed, revegetation is a critical measure for soil and water conservation engineering (Heath et al., 2005; Yu et al., 2017; Bai et al., 2022). The abandonment of farmland and revegetation can enhance soil porosity and soil organic matter content and lower bulk density, which affect soil nutrient contents, permeability, respiration, root biomass, microbial composition, and activity (Zhao et al., 2013; Guo et al., 2020; Liu et al., 2020; Post and Knapp, 2021; Wu et al., 2021).

Soil nutrients have substantial effects on plant growth and play important roles in productivity and ecosystem functions (Zhang et al., 2022). Soil carbon is essential for maintaining soil nutrients, which influence soil biotic and abiotic properties (Ge et al., 2020; Cheng et al., 2021; Lyu et al., 2022). Soil nitrogen, phosphorus, and potassium are three important elements that individually or jointly affect plant resilience and stability (Guan et al., 2017; Duan et al., 2020; Yang et al., 2020; Salekin et al., 2021). Numerous studies of the effects of soil properties on plant growth have demonstrated that revegetation is threatened by the spatial heterogeneity of soil salinization, indicating that soil conditions have a significant impact on plant growth (Hao et al., 2010; Huang et al., 2019; Han et al., 2020; Wang et al., 2021a). The distribution variability of soil properties is likely to be a critical driving force in forming vegetation distribution patterns and adaptation strategies (Li et al., 2020; Zhang et al., 2020b; Wang et al., 2021b). Recent research found that soil nitrogen, soil phosphorus, and soil potassium concentrations in arid regions decreased significantly with increased groundwater depth, aggravating erosion (Zhang et al., 2018; Huang et al., 2019; Wang et al., 2021b). These results emphasize that it is requisite to enhance the comprehension of the connection between soil, vegetation, and alteration in groundwater depth in arid and semi-arid ecosystems.

Horqin Sandy Land is a representative and sensitive ecological region located in the agropastoral transitional zone between the Inner Mongolian Plateau and the Northeast Plains (42°41′–45°45′ N and 118°350–123°30′ E) and one of the four largest sandy lands in northern China. It covers an area of approximately 139,300 km^2^, which has been desertified into a sandy land area of up to 71,884 km^2^ (Wang, 2016; Luo et al., 2017; Zuo et al., 2020). The landscape in this area is characterized by sand dunes that alternate with gently undulating lowland areas (Luo et al., 2020). This area belongs to the continental semiarid monsoon climate and is in the temperate zone, with a mean annual temperature of 3–7°C and a mean annual rainfall (AP) of 350–500 mm (Huang et al., 2022). In recent years, many ecological problems have been caused by rapid population growth and increased demand for land, food, housing, and employment (Kooch et al., 2022), especially the decline of the groundwater level (Maihemuti et al., 2021). At the same time, the aggravation in evapotranspiration would be more evident than rainfall with climate warming in the future, which would result in more drought and impulse the aridification of this area (Dai and Zhao, 2017; Su et al., 2018). These various climatic conditions may be expected to enhance the vulnerability of sand ecosystems and may produce strong impacts on the biotic and abiotic processes of native vegetation in the future. More relevant studies have observed or experimentally determined how hydropenic stress affects vegetation communities, but the majority of approaches have only researched upper soil drought that is caused by shallow groundwater depth and precipitation deficits (Germino and Reinhardt, 2014; Awad et al., 2018; Kulmatiski et al., 2020; Griffin-Nolan et al., 2021). However, in a ‘deep’ drought that is determined by changes to the deep groundwater depth, plants may preferentially seek available soil water in deeper soil by adjusting community characteristics (Comas et al., 2013; Mengistu et al., 2021; Lozano et al., 2022). There is less research on the influence of deeper groundwater depth on soil properties. Hence, the objectives of this study were to systematically analyze the traits of vegetation communities and the distribution of soil resources with deep groundwater depth in the agro-pasture crisscross region of the Horqin Sandy Land, to analyze the response of soil and plants under various deep underground water tables, and to provide support for revegetation and the conservation of groundwater. Our research is seeking to explore the following objectives: (1) investigate the vegetation community traits in relation to different deep groundwater depths in three successional stages; (2) analyze the spatial distribution of soil properties in 0–200 cm soil layers under different deep groundwater depth in the three successional stages; and (3) evaluate the relative contribution of deep groundwater depths to the vegetation community characteristics and soil nutrients distribution and their coupled relationship among the three.

## 2 Materials and methods

### 2.1 Experimental site

The study was conducted at the Naiman Desertification Research Station (42° 58′ N and 120° 43′ E), Chinese Academy of Sciences, which is in the southeast of Horqin Sandy Land, eastern Inner Mongolia, China. The study area belongs to the typical temperate semiarid continental monsoon climate. The average annual precipitation was 351.7 mm (Huang et al., 2022), with an uneven spatial and temporal distribution, in which the precipitation from June to September accounted for about 80%. The mean annual temperature is 5.8–6.4°C.

### 2.2 Experimental set-up and sampling

Soil and vegetation were sampled in August 2021, during the peak period of plant growth. Based on the vegetation coverage and degree of soil surface fixation in the Horqin sandy land (Zuo et al., 2010), three dune types (fixed, semi-fixed, and mobile dunes) were decided, where arbor (*Ulmus pumila L.*, *Pinus sylvestris* var. *mongholica Litv.*) and shrub (*Artemisia halodendron Turcz. et Bess.*, *Caragana microphylla Lam.*) were the dominant species, joined by preponderant herbs (*Pennisetum centrasiaticum Tzvel.*, *Setaria viridis (L.) Beauv.*, *Cleistogenes squarrosa (Trin.) Keng*, *Leontopodium leontopodioides*, *Corispermum hyssopifolium L.*, and *Tribulus terrestris L.*). The mobile dune (MD), which had 10%–30% vegetation coverage and >70% mobile sand, is an early successional stage. The semi-fixed dune (SFD), which had 30%–60% vegetation coverage and >10% mobile sand, is a medium-successional stage. The fixed dune (FD), which had >60% vegetation coverage and no mobile sand, is a late-successional stage. Each dune type was represented by one successional stage; detailed site information is shown in **Table 1**. Three plots (10 m × 10 m, >10 m apart) with six quadrats (1 m × 1 m) were established on each dune with different groundwater depths (6.25 m, 10.61 m, and 15.26 m). Soil and vegetation samples were gathered at these locations in each plot, with 6.25 m, 10.61 m, and 15.26 m of groundwater depth. Herbs and shrubs are dominant components in sandy systems in this research, so we aimed to explore the characteristics of vegetation communities. In each 1 × 1 m quadrat for the vegetation community, we investigated coverage, abundance, and average height. For species with clonal growth, we visually considered each clump as one ‘individual’ (Cheplick and Clay, 1989), with the coverage estimated as an ellipse equation using the length of the longest axis and the length of the axis perpendicular to the longest axis. The aboveground leaves were then clipped, sorted by species, and dried at 60 °C for 48 h to estimate chemical properties. A total of 108 leaf and soil samples were collected (six vegetation quadrats per plot × three dunes × three groundwater depth plots per dune). Soil samples are removed from roots, litter, and gravel by passing through a 2-mm mesh sieve and being divided into two subsamples. Then we use one subsample to measure soil moisture immediately, and the other one is air-dried for physicochemical analysis.

**Table 1:** Investigative sites information and the dominant vegetation communities.

Groundwater Depth (m)	Successional Stage (dune)	Vegetation Types	Typical Communities
6.25	MD	Shrubs/Herbs	*Caragana microphylla/Pennisetum centrasiaticum*
	SFD	Shrubs/Herbs	*Caragana microphylla/Setaria viridis*
	FD	Trees/Shrubs/Herbs	*Ulmus pumila/Pinus sylvestris/Artemisia halodendron/Pennisetum centrasiaticum*
10.61	MD	Shrubs/Herbs	*Caragana microphylla/Pennisetum centrasiaticum*
	SFD	Trees/Shrubs/Herbs	*Ulmus pumila/Pinus sylvestris/Artemisia halodendron/Pennisetum centrasiaticum*
	FD	Trees/Shrubs/Herbs	*Ulmus pumila/Pinus sylvestris/Artemisia halodendron/Pennisetum centrasiaticum*
15.26	MD	Trees/Herbs	*Pinus sylvestris/Pennisetum centrasiaticum*
	SFD	Shrubs/Herbs	*Artemisia halodendron/Pennisetum centrasiaticum*
	FD	Trees/Shrubs/Herbs	*Ulmus pumila/Pinus sylvestris/Artemisia halodendron/Pennisetum centrasiaticum*

### 2.3 Measurement of plant and soil properties

For plants, five functional traits were chosen from two trait categories (vegetation community morphology and whole-plant chemical traits), including average height, species abundance, community coverage, leaf total nitrogen (LN), and leaf total carbon content (LC). These traits were measured according to the protocol described by Perez-Harguindeguy et al. (2013). We measured these five functional traits for the community of investigative species, and these species accounted for more than 90% of the total local species. Soil water content (SWC) was measured gravimetrically by oven-drying at 105°C to a constant weight. And soil pH was determined in a 1:1.5 soil–water extract using a pH probe. Soil organic carbon (SOC) was measured by the dichromate oxidation method. Soil total nitrogen (STN) was measured by the Kjeldahl acid-digestion method with an Alpkem autoanalyzer. Soil variables included TC, SOC, TN, AN, AK, AP, soil pH, and soil water content.

### 2.4 Data analysis

First, soil properties, vegetation morphological traits, and community characteristics (Shannon index and Simpson index) were compared among different groundwater depths and successional stages by using the “aov” function to perform ANOVAs. When ANOVAs showed significance, the means among groundwater depths or successional stages were compared with Tukey HSD *post hoc* tests. To disentangle the significant effects of different groundwater depth levels in the three successional stages, we used the pairwise PERMANOVA method by using the ‘adonis’ function in the “vegan” package (Oksanen et al., 2007). The Mantel test was used to analyze the correlations between soil properties and beta diversity (community composition) of plant communities using the “vegan” package (Oksanen et al., 2007). All statistical analyses were carried out using R version 4.2.0 (Team R. C, 2017).

## 3 Results

### 3.1 Vegetation community traits

**Figure 1:**
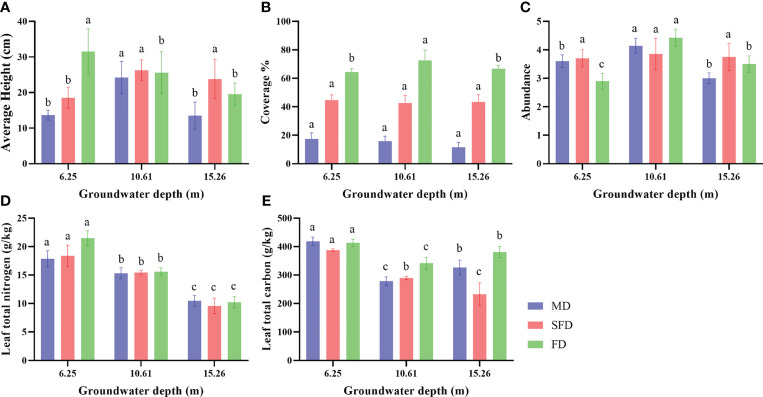
Barplot of plant growth parameters in different groundwater depth (GWD) observed between fixed dune (FD), semi-fixed dune (SFD) and moving dune (MD). The three groundwater depth (GWD) treatments include 6.25 m, 10.61 m and 15.26 m treatments. **(A)** Average height, **(B)** Coverage, **(C)** Abundance, **(D)** Leaf total nitrogen, **(E)** Leaf total carbon. Letters denote significant differences between GWD treatments (estimated marginal means, *p*<0.05).

This result showed that all morphological and physicochemical traits were significantly impacted by groundwater depth in different dune types (**Figure 1**). Different GWD levels altered the plant average height in the fixed dune. When GWD was elevated, the average height of vegetation was significantly reduced (*p* <0.05), and their average height ranged from 19.54 cm to 31.51 cm, which was greater than that in SFD and MD (**Figure 1A**). Vegetation coverage responses to altered GWD were different between the three dune types. In the fixed dune, coverage increased first and then declined with increasing GWD (*p* <0.05); however, in the semi-fixed dune and moving dune, there was no significant difference among GWD levels (**Figure 1B**). Species abundance was significantly different among GWD levels in FD and MD (*p* <0.05) and suggested a tendency to increase first and then decrease with increasing groundwater depth (**Figure 1C**). The biochemical traits were pronounced under the groundwater depth treatments. GWD levels effectively affect the content of leaf nitrogen (LN) and leaf carbon (LC). As shown in **Figure 1D**, the LN was significantly reduced by increasing GWD levels at each successional stage (*p* <0.05), and their LN content ranged from 10.24 g/kg to 21.49 g/kg. As shown in **Figure 1E**, the LC was significantly reduced by increasing GWD levels in SFD (*p* <0.05), and their LC content ranged from 232.83 g/kg to 387.59 g/kg. In FD and MD, LC decreased first and then increased with the increase of GWD (*p* <0.05), while the LC contents corresponding to FD and MD ranged from 380.91 g/kg to 413.44 g/kg and 326.75 g/kg to 418.66 g/kg, respectively.

**Figure 2:**
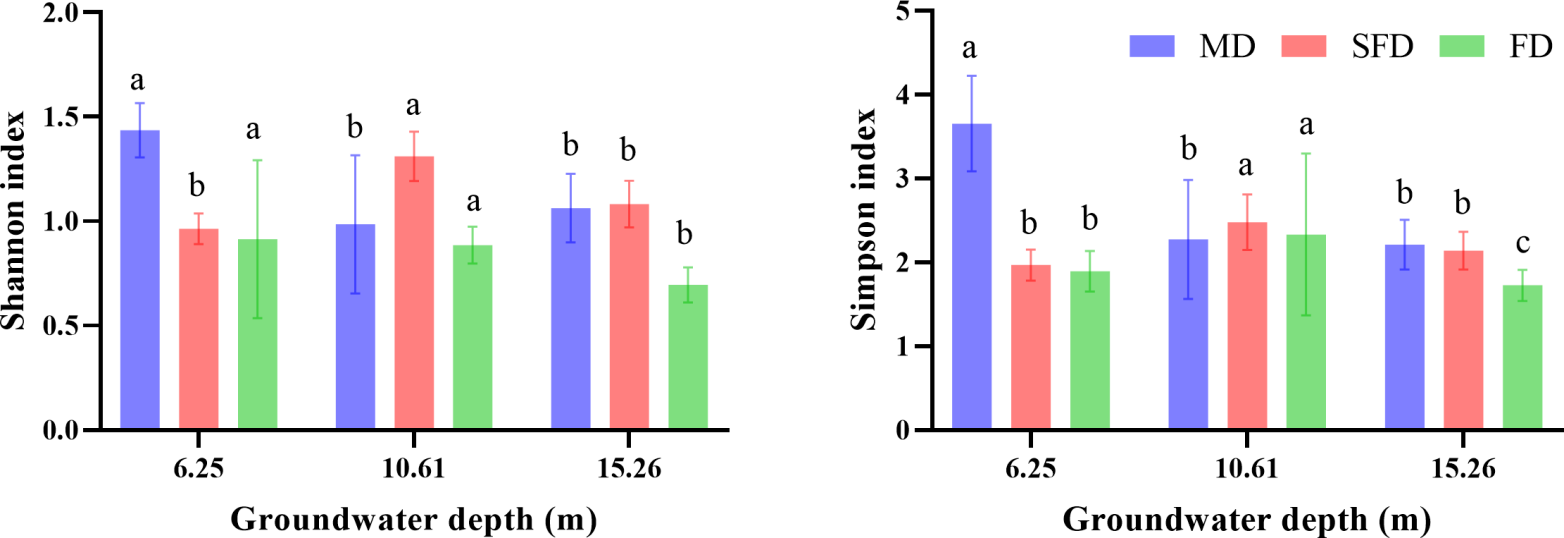
Boxplot of diversity in different groundwater depth (GWD) experiments in three dune types. The three GWD treatments include 6.25 m, 10.61 m, and 15.26 m. Letters denote significant differences between GWD treatments (estimated marginal means, *p* >0.05).

The diversity index was significantly affected by groundwater depth, as shown in **Figure 2** (*p* <0.05). MD had the highest Shannon and Simpson index at 6.25 m groundwater depth, whereas FD and SFD had the highest Shannon and Simpson index at 10.61 m groundwater depth. The general trend of the diversity index decreased with increasing groundwater depth.

### 3.2 Sandy soil profile properties

**Figure 3:**
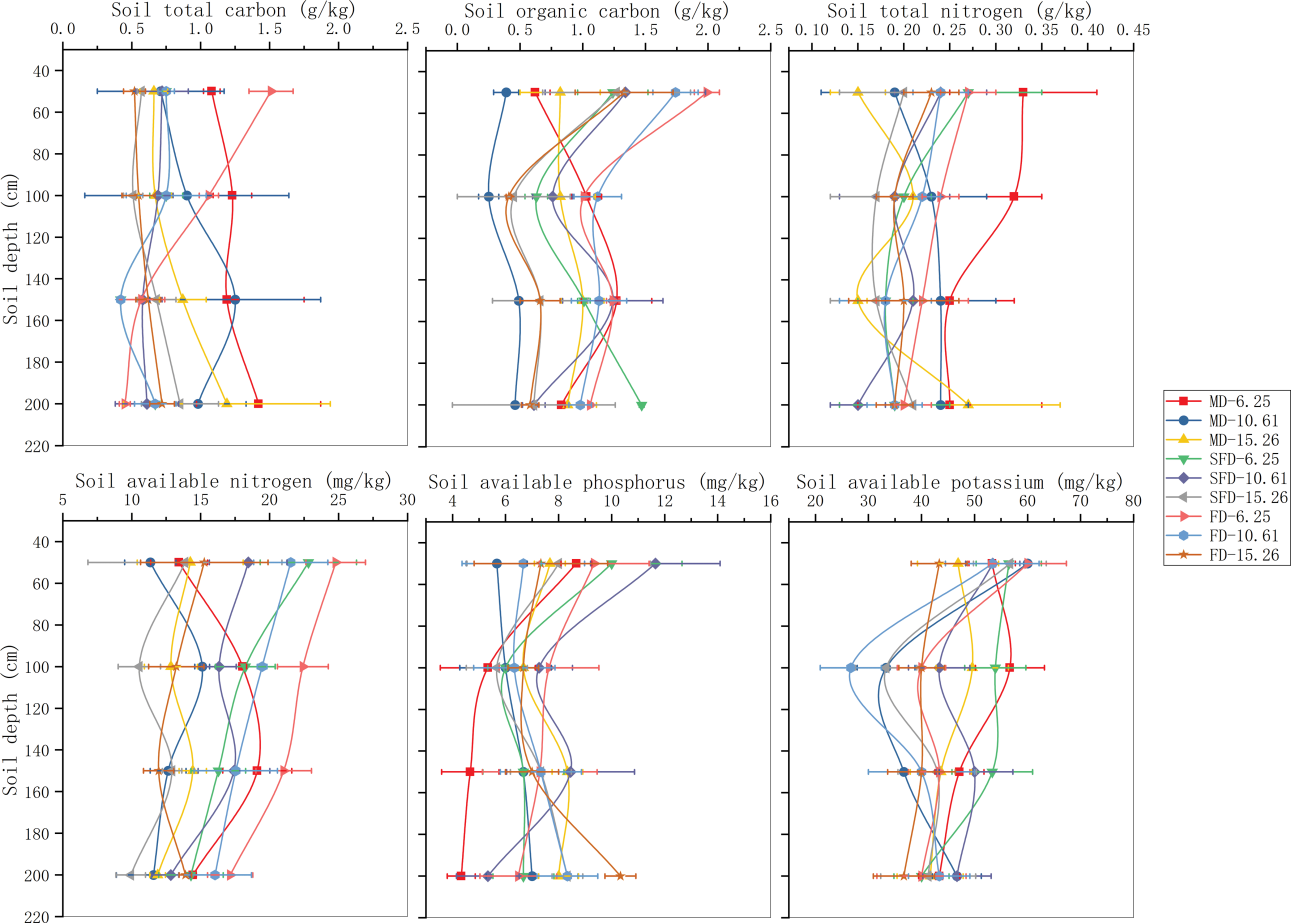
Chemical properties of different soil profile depths in different groundwater depths in different types of dunes.

The chemical properties of soil in different dunes showed significant changes with groundwater depth (**Figure 3**). The content of TC in FD was higher than that in MD and SFD, and particularly higher in the shallower groundwater depth treatment as compared to the deeper groundwater depth treatments. Changes in the content of the TC were also observed with increased soil depth. The TC decreased with an increase in soil profile in FD and SFD and was marginally higher at the bottom layer as compared to that of the top layers in MD. Similarly, the SOC showed an analogical trend in different dune types with TC, but the concentration of SOC decreased with an increase in groundwater depth, especially when the difference was stronger between FD and SFD. Meanwhile, SOC decreased with a deeper soil profile.

The content of TN in FD was also significantly higher than that at SFD and MD, and TN in different dunes showed larger contents at the middle groundwater depth (10.61 m) as compared to the shallower and deeper depths of groundwater. The TN increased with deeper soil depth in MD, whereas there was an opposite trend in FD and SFD, and the tendency of the TN value was consistent with that of TC. AN in FD showed higher contents as compared to SFD and MD. The changes with groundwater depth also indicated a significant difference, which had the highest AN at 10.61 m groundwater depth. The content of AN with deeper soil depth decreased as a whole. AP in FD was particularly higher as compared to SFD and MD, and the changes in AP showed a decline, followed by a GWD increase. The AP decreased with deeper soil depth in general. AK differed due to different groundwater depths among successional stages, which were strongest in MD, then SFD, and eventually FD. The content of AK decreased first and then increased with a declining groundwater table. AK decreased in total with the increase of soil depth.

**Figure 4:**
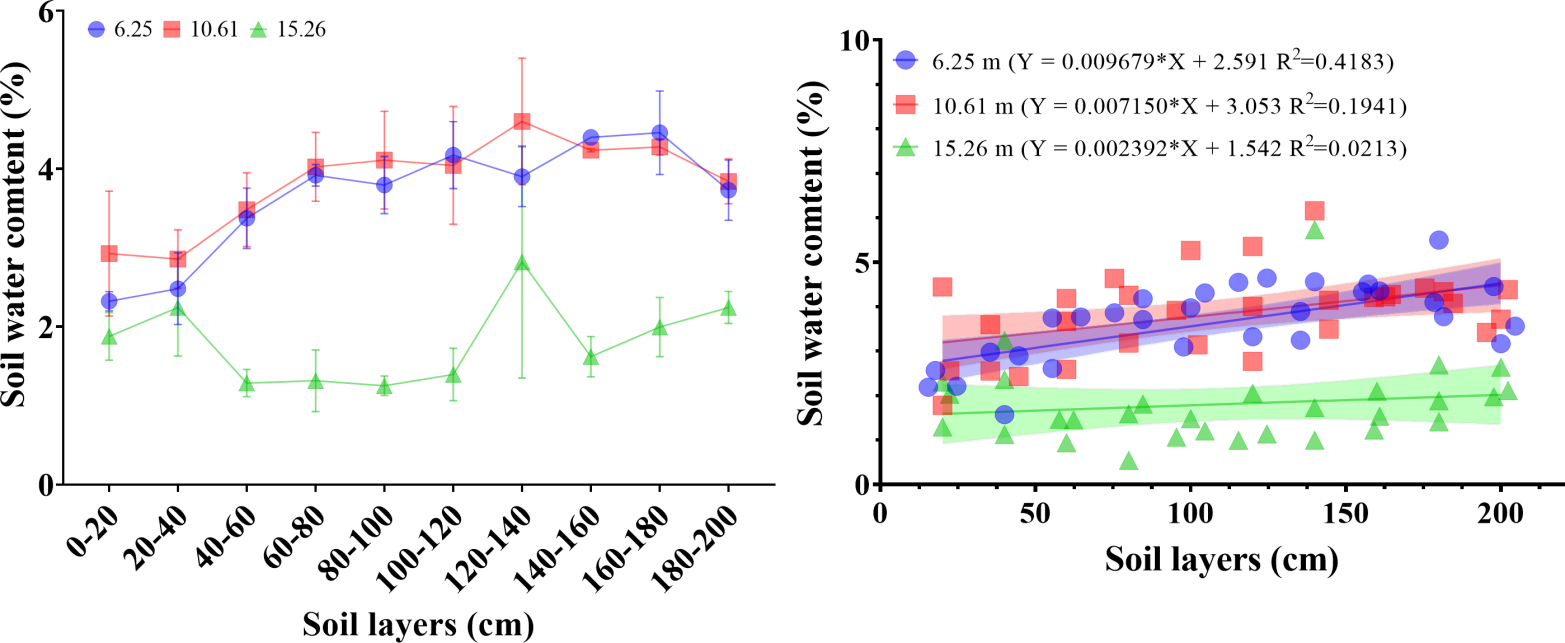
The effects of groundwater depth change on the water content of the soil profile (0–200 cm). (D) Groundwater depth was 15.26 m; (M) Groundwater depth was 10.61 m; and (S) Groundwater depth was 6.25 m.

Groundwater depth and soil profile treatment altered soil moisture (**Figure 4**). The soil water content of 0–200 cm soil layers in the same site was affected by soil depth, and the water content ranged from 2.32% to 4.45%, 2.86% to 4.61%, and 1.25% to 2.82% in sites with 6.25 m, 10.61 m, and 15.26 m groundwater depth, respectively. When groundwater depth increased, soil water content reduced, but increased in the 0–20 cm soil layer first, then declined. From the soil profile perspective, soil moisture increased with increasing soil depth in 0–80 cm soil layers at 6.25 m and 10.61 m groundwater depths. Then soil water content remained relatively stable in the 80–180 cm soil profile and declined in the 200 cm soil layer. Compared with the sites of 6.25 m and 10.61 m groundwater depth, soil moisture exhibited a relatively stable trend with increasing soil depth, except for an elevation in soil water content in the 120–140 cm soil profile at 15.26 m groundwater depth.

### 3.3 Sandy soil properties

**Figure 5:**
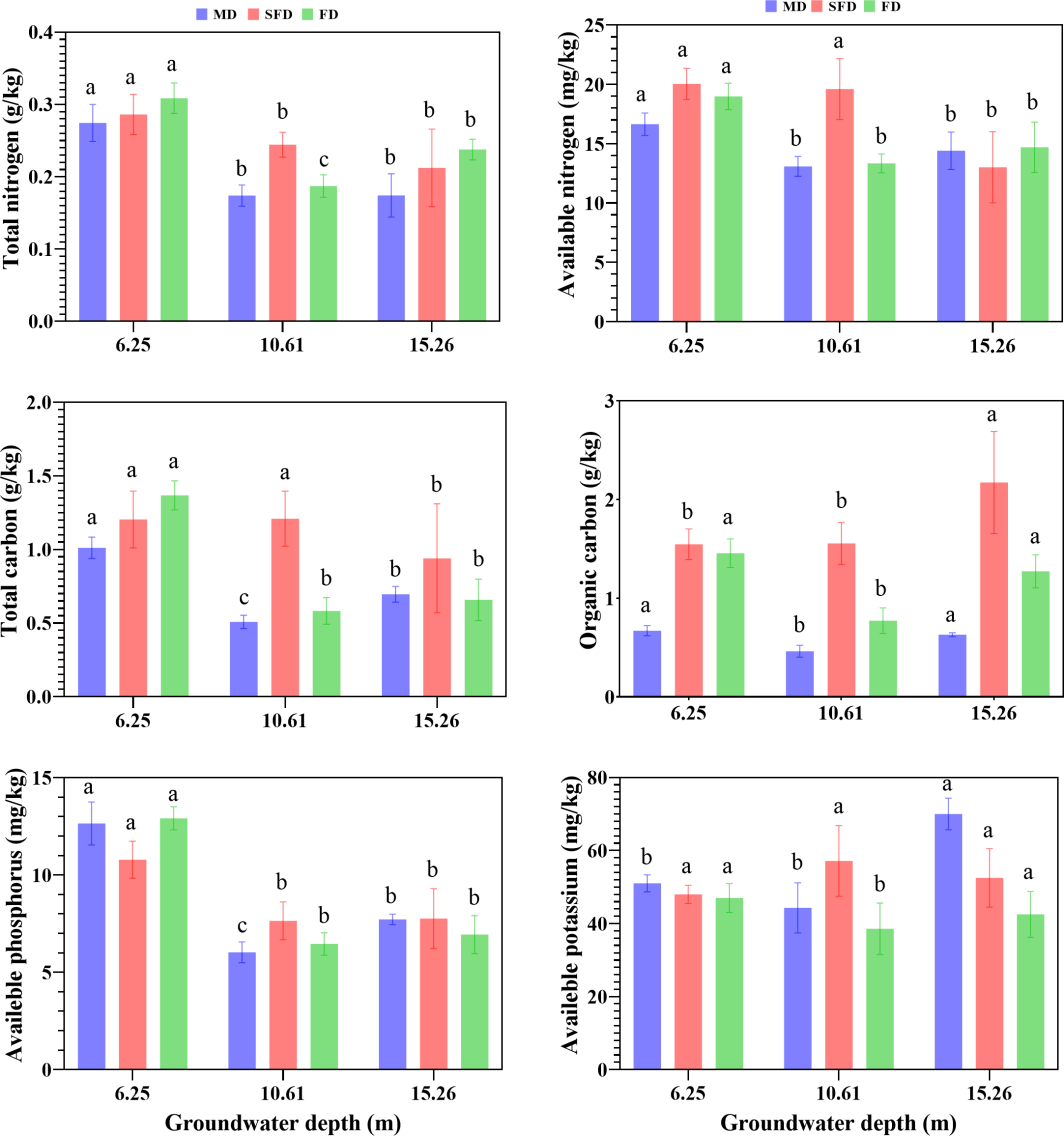
Barplot of soil nutrients at different groundwater depths (GWDs) in fixed dune (FD), semi-fixed dune (SFD), and moving dune (MD). The three-groundwater depth (GWD) observation sites include 6.25 m, 10.61 m, and 15.26 m treatments. Letters denote significant differences between treatments (estimated marginal means, *p* <0.05).

Deep groundwater depth and successional stages cause variations in soil properties (**Figure 5**). The content of STN was higher in FD as compared to SFD and MD and significantly reduced with increasing groundwater depth (*p* <0.05), which ranged from 0.17 g/kg to 0.31 g/kg. Soil available nitrogen (AN) was higher in SFD than that in FD and MD and significantly declined with groundwater depth (*p* <0.05), especially in FD (13.32–18.97 mg/kg). The content of STC was higher in SFD than in FD and MD. In the same dune area, increasing groundwater depth led to decreased STC content (*p* <0.05). The content of SOC significantly declined first and then increased with increasing groundwater depth in FD and MD (*p* <0.05), and the STC content in SFD was greater than that in FD and MD. The content of AP significantly decreased with increasing groundwater depth among the three successional stages (*p* <0.05). The content of AK significantly increased with groundwater depth in MD (*p* <0.05), which ranged from 44.29 mg/kg to 69.83 mg/kg. AK content particularly decreased first and then increased with increasing groundwater depth in FD (*p* <0.05). But there were no significant differences among groundwater depths in SFD.

**Figure 6:**
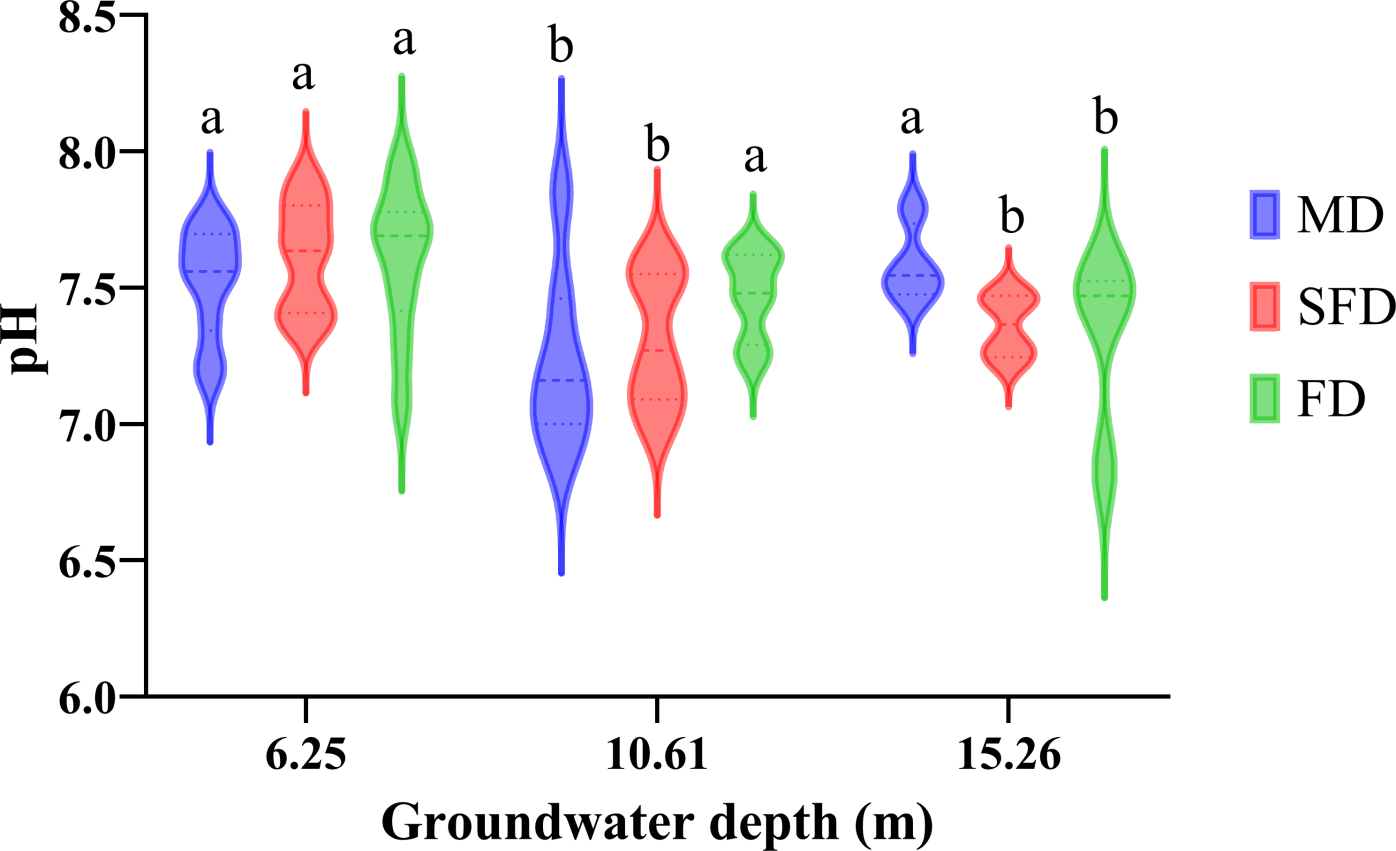
Boxplot of soil pH at different groundwater depths (GWDs) observed in both dune types. The three GWD treatments include 6.25 m, 10.61 m, and 15.26 m. Letters denote significant differences between treatments (estimated marginal means, *p* <0.05).

As shown in **Figure 6**, soil pH was significantly affected by groundwater depth among the three successional stages. In FD, soil pH significantly decreased first and then increased with increasing groundwater depth (*p* <0.05) and ranged from 6.83 to 7.96. Generally, soil pH significantly decreased with groundwater depth except for FD (*p* <0.05), and the soil pH ranged from 6.87–7.85 and 6.98–7.91 in MD and SFD, respectively.

### 3.4 Responses of plant and soil characteristics to successional stage and groundwater depth

**Figure 7:**
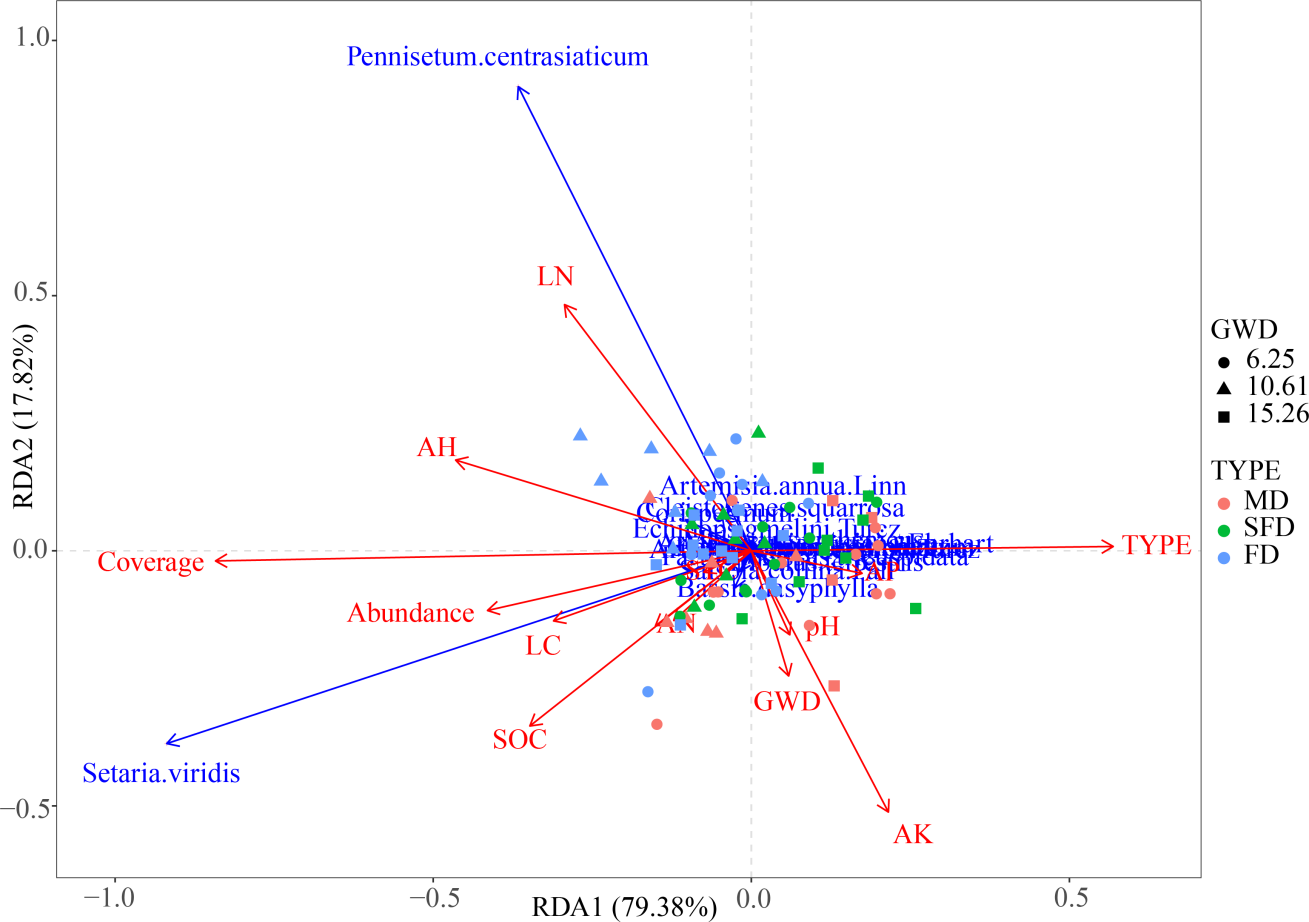
Redundancy analysis (RDA) of environmental variables and 54 vegetation quadrat data (species abundance) from nine investigated sites in the Horqin Sandy Land, northeastern China.

Redundancy analysis was used to analyze the relationship between environmental conditions and species distribution. The first two axes explained a total of 97.20% of the variation in species distributions, of which the first axis explained 79.38% and the second 17.82% (**Figure 7**). Based on RDA output, there was a negative correlation between groundwater depth and average height, cover, species abundance, PTN, PTC, AN, and SOC. It was found that pH, AP, and AK were positively correlated with groundwater depth. We also found that type (successional stage) was affected by soil nutrients and vegetation community characteristics, including PTN, PTC, SOC, AN, average height, species abundance, and coverage. Besides, the results also showed that *P. centrasiaticum Tzvel.* and *S. viridis (L.) Beauv.* were mainly distributed in areas with lower underground water tables, higher coverage, soil organic carbon, species abundance, and soil nitrogen. The distribution of the arbor and shrub species was sparse in places with higher groundwater depths, especially *U. pumila L.*, *P. sylvestris* var., *mongholica Litv.*, *A. halodendron Turcz. et Bess.*, *and C. microphylla Lam*.

**Figure 8:**
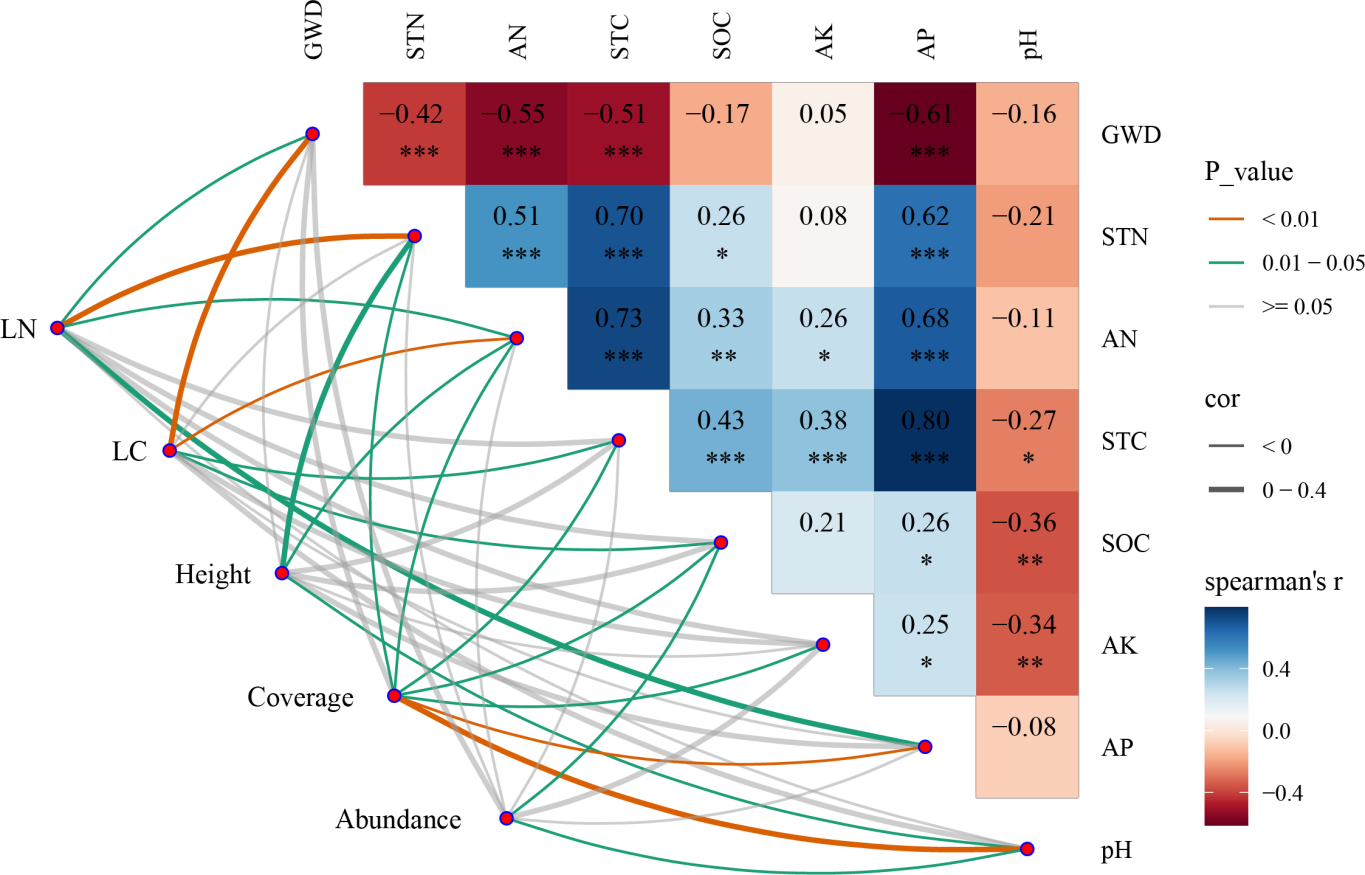
Relationships between vegetation community characteristics and soil properties by the Mantel test.

The Mantel test revealed that plant growth parameters were significantly affected by soil properties and groundwater depth (**Figure 8**). Pearson correlations showed that LN (leaf total nitrogen) had a significant correlation with GWD, STN, AN, and pH (*p* <0.05). LC (leaf total carbon) was significantly related to GWD, AN, STC, and SOC (*p* <0.05). The vegetation average height had a significant correlation with STN, AN, and AP (*p* <0.05), and the vegetation coverage significantly correlated with GWD, STN, AN, STC, SOC, AK, and AP (*p* <0.05), while the species abundance had a significant correlation with SOC and pH (*p* <0.05), and not significant correlation with GWD and other soil properties. GWD significantly affected STN, AN, STC, and AP (*p* <0.05), the correlation between them was −0.42, −0.55, −0.51, and −0.61, respectively.

## 4 Discussion

### 4.1 Vegetation traits respond to dune types and continual increases in groundwater depth

Our results suggested that the morphological characteristics of the vegetation community showed strong heterogeneity in response to alterations in three types of dunes with different groundwater depths, and this was not evident for average height and species abundance in successional stages (**Figure 1**). Average height and species abundance were the highest at 10.26 m groundwater depth, and it may be due to the soils at 10.26 m groundwater depth having better soil conditions. Because previous studies have reported that plant communities are tightly coupled with hydrological processes, the ideal groundwater depth for plant growth is not the lowest groundwater table (Gorai et al., 2010; Zhu et al., 2012; Sun et al., 2020; Bai et al., 2021; Sun et al., 2022). Wang obtained different results and demonstrated that the growth and abundance of vegetation communities will decrease at groundwater depths greater than 6 m due to herb vegetation that could hardly exist (Wang et al., 2021c). These discrepancies between the two study areas may be due to differences in climate or vegetation species and characteristics. For plant chemical traits, leaf total nitrogen and carbon showed significant variation at different groundwater depths among the successional stages. Leaf total nitrogen contents were greater at 6.25 m groundwater depth than at 10.61 m and 15.26 m groundwater depth in this study (**Figure 1D**). Plant total carbon contents were greater at 6.25 m groundwater depths than at 10.61 m and 15.26 m groundwater depths. It is attributed to the dominant species of shrubs, which have deep roots (Gorai et al., 2010; Fu et al., 2014; Wang et al., 2021d).

### 4.2 Properties of soil profile in sandy land

Successional stages and groundwater depths affected spatial differences in the properties of the soil profile. **Figure 3** shows that the nutrient contents of the soil profile were greater in FD and SFD, while the nutrient leaching effect was stronger in MD than in FD and SFD. Previous studies have suggested that soil texture improves better in the later successional stage, which is affected by vegetation growth and soil porosity (Guzman et al., 2019; Zheng et al., 2021). Meanwhile, groundwater depth also influences nutrient accumulation in the soil layers (Su et al., 2014; Zhang et al., 2020a). In this study, soil nutrient concentrations were higher in the upper soil, and these concentrations showed a gradual decrease towards the groundwater interface, but the change in the TC concentration was opposite in MD. The deeper the groundwater depth, the easier it is for nutrients to accumulate in the unsaturated soil profile above the water table (Rasiah et al., 2013). The present study also showed that the contribution of vegetation growth to soil nutrients gradually declined with soil depth, but the contribution of groundwater depth improved in soil layers (Yu et al., 2020). A previous study showed that the essential driving force for controlling the overall ecosystem may be the dynamics of available water relative to groundwater depth (Zhang et al., 2018; Sun et al., 2020; Chen et al., 2021; Wang et al., 2021d). Several results reported that soil moisture is affected primarily by altered groundwater depth because of high evaporation and few precipitation events in arid and semi-arid regions (Yu et al., 2013; Karthe, 2018; Rivas et al., 2020; Chen et al., 2021). Our results showed that soil moisture along the 0–200 cm soil layers gradually increased with increasing soil depth at 6.25 m and 10.61 m groundwater depths, but the soil moisture along the 0–200 cm soil depth at 15.62 m groundwater depth did not show a significant trend with increasing soil depth. Meanwhile, soil moisture decreased with increasing groundwater depth in total, and this result is consistent with many other studies. These results indicated that the discrepancy of groundwater depth in specific terrains may form a specific microclimate or habitat traits that regulate vegetation growth and comprise a specific microenvironment, with the addition of desorption, dissolution, or the lateral export of groundwater, ultimately acting on the accumulation and distribution of nutrients in different profiles of soils (Shi and Shao, 2000; Wang, 2002; Xu et al., 2012; Yu et al., 2019). Several studies suggested that precipitation was the driving factor of nutrient distribution in soil profiles at global, regional, and site scales (Zhang et al., 2019; Yu et al., 2022). However, in arid and semi-arid regions characterized by scarce rainfall and intense evaporation, groundwater serves as a vital and sole source of water, and the variability of soil moisture primarily depends on groundwater depth. The dynamic fluctuations of soil moisture are considered the most significant driving force controlling the entire ecosystem in this region (Stirzaker et al., 1996; Fu et al., 2014). The impact of successional stages on soil nutrients may be diluted by the effects of altered groundwater depth.

### 4.3 Soil properties in sandy land

More studies reported that the distribution of soil nutrients is influenced by relevant elements and complicated regulating mechanisms, as well as human activities, various location conditions, and scales (Ge et al., 2020). In arid and semi-arid regions, soil moisture is an essential driving factor that transports and stores nutrients for plant growth (Yu et al., 2013; Chen et al., 2021). Hydraulic lifting enables plant roots to acquire their necessary water from deeper soil layers and groundwater (Prieto et al., 2012). Therefore, soil nutrients might be affected by the impact of chemical, physical, and biotic factors related to groundwater depth. Our results showed that deeper groundwater had a strong effect on soil total carbon, total nitrogen, available nitrogen, and available phosphorus contents. Previous studies reported that groundwater depth affects the soil nitrogen concentration, and the soil nitrogen concentration decreased with increasing groundwater depth (Zhang et al., 2022). Our finding was related to the fact that the extent of soil nitrogen concentration that leached to the groundwater interface decreased with increasing depth, and this result is in accordance with many other studies (Granlund et al., 2000). Soil available phosphorus concentration significantly declined with an increase in groundwater depth, and it was consistent with the result that groundwater depth had an obvious impact on soil phosphorus concentration within deep groundwater extents (Wu et al., 2014; Zhang et al., 2018). Chen concluded that soil organic carbon and soil available potassium concentrations at deep groundwater depth were higher than those at shallow groundwater depth (Chen et al., 2021). According to a previous study, the surface salinization of soil and the degree of groundwater mineralization increase with the rise in the underground water table. When dissolved salts from groundwater rise to the soil surface and accumulate with soil water evaporation, soil salinization occurs (Orellana et al., 2012). When the groundwater depth ranges from 1 to 2 m, the increased soil capillary water content intensifies the evaporation induced by solar radiation, thereby exacerbating surface soil salinization. When the groundwater depth ranges from 2 to 4 m, the soil capillary water table decreases, reducing the evaporation of groundwater into the atmosphere and lowering the soil salt content. When the groundwater depth exceeds 4 m, the lowered underground water table impairs the soil capillary water evaporation effect in the surface soil, resulting in a significant decrease in surface soil salt content (Maihemuti et al., 2021). However, Hu discovered that intense evaporation caused by low vegetation coverage promotes salt accumulation in the surface soil, leading to a decrease in dissolved salt content and an increase in soil pH through desalination exchange and alkalinization processes in the region characterized by uneven rainfall (Hu et al., 2021). In this research, the soil pH declined with increasing groundwater depth in the three successional stages, and this was consistent with other studies showing that strong evaporation, which enhanced salt accumulation at the soil’s upper layers, resulted in a high pH (Li et al., 2013).

### 4.4 Relationship among soil, plant, and groundwater depth

For arid and semi-arid regions, groundwater was the key source of water for plant growth, while large natural vegetation species in this area were lost due to excessively extracted groundwater, which led to groundwater levels persistently decreasing (Su et al., 2021; Trinidad Torres-Garcia et al., 2022). Our findings showed that deep groundwater depth was one of the most critical environmental elements influencing soil and plants in Horqin Sandy Land, and soil moisture is an increasing limiting factor for plant growth (Prieto et al., 2012). Several studies in arid and semi-arid regions demonstrated that the effect of deeper groundwater depth on shrubs was greater than that on herbs, and related to the water use strategies of different plants (Zhang et al., 2018; Imin et al., 2021). This finding supports our results that the *P. centrasiaticum Tzvel.* and *S. viridis (L.) Beauv.* were mainly distributed in areas with lower underground water tables, whereas the distribution of the arbor and shrub species is sparse in regions with deeper groundwater tables, especially *U. pumila L.*, *P. sylvestris* var., *mongholica Litv.*, *A. halodendron Turcz. et Bess.*, and *C. microphylla Lam.*, manifesting strong drought tolerance. Consequently, plants have adaptive strategies to enhance necessary resource utilization efficiency to accommodate environmental changes in arid and semi-arid regions, and there are also opportunists that have relatively wide niches (e.g., *Phragmites australis* and *Tamarix ramosissima*), commonly with higher SLA and leaf nitrogen content, and survive along roadsides, salt marshes, and more hostile environmental areas (Chen et al., 2021). Meanwhile, vegetation restoration has a positive impact on soil properties, contributes to circulation of soil nutrients, and improves soil properties beneficial to plant growth (Feng, 1998; Kardol et al., 2006; Ding and Eldridge, 2021). These results demonstrated that vegetation–soil feedback could affect community structure and soil condition, improving water use efficiency to some extent.

Although we did a series of research studies through field observation, there are still the following limitations to our study: First, we just tested the plant and soil properties in relation to groundwater depth at 6.25 m, 10.61 m, and 15.26 m because this range of groundwater depth is present in the current study area. Second, the impact of groundwater depth on plants and soil could be discerned and estimated over the long term, and it is a complicated process. Therefore, more elements of influence and long-term observation data on plants and soil in the field are needed now and in the future. Anyhow, our research had observed and summarized the investigation of the variation in plants and soil in relation to deep underground water change in Horqin Sandy Land and laid down a supporting basis for breaking the above limitations.

## 5 Conclusions

In summary, our results suggested that deeper groundwater depth affected vegetation and soil properties, either directly or indirectly. The most essential traits for vegetation communities, including coverage, average height, species abundance, Shannon index, and Simpson index of vegetation diversity, are indicative of environmental changes. Most soil properties were suggested to change from enrichment to barren, such as STN, AN, and STC, with the increase in groundwater depth, while soil pH, SOC, and AK respond in the opposite way. Soil moisture was found to increase first and then decline with an increase in soil depth, especially in the response of soil moisture at 6.25 m and 10.61 m groundwater depths. Overall, soil moisture decreased with increasing groundwater depth. Correlation analysis showed different responses for arbors/shrubs compared to herbs, reflecting discrepancies in their adaptability to environmental stresses. Changes in groundwater depth and successional stages were critical driving factors in species distribution. Compared to herbs, arbors and shrubs exhibited stronger adaptability to environmental changes such as deeper groundwater depth. The plasticity of arbor and shrub species to alterations in deeper groundwater depth may alleviate the negative impacts of environmental stresses. Therefore, groundwater depth is one of the most important factors for vegetation and soil in semi-arid ecosystems, and this research is helpful to the restoration of degraded vegetation and soil by providing theoretical and empirical support for adaptive plant species arrangement and sustainable water use in the region and elsewhere.

## Data availability statement

The original contributions presented in the study are included in the article/supplementary materials. Further inquiries can be directed to the corresponding authors.

## Author contributions

SZ: Conceptualization, data curation, formal analysis, investigation, methodology, validation, visualization, writing—original draft, writing—review and editing. XZ: Funding acquisition, project administration, resources, supervision, validation, visualization, writing—original draft, writing—review and editing. YL: Conceptualization, formal analysis, investigation, methodology, resources, supervision, validation, visualization, writing—original draft, writing—review and editing. XC: Formal analysis. CL: Formal analysis. HF: Formal analysis. WL: Formal analysis. WG: Formal analysis. All authors contributed to the article and approved the submitted version.
